# A Conversation with Stephen Cochrane

**DOI:** 10.1021/acscentsci.3c00782

**Published:** 2023-07-10

**Authors:** Geoffrey Kamadi

Antibiotics researchers have long focused on the proteins inside bacteria, hoping to find a key piece of the bacteria’s molecular machinery that, when disrupted, kills the bugs and cures infections. But that process requires that antibiotic molecules cross the bacteria’s cell membrane to reach those targets. Once inside, the antibiotics face a slew of defenses that bacteria have evolved to neutralize their would-be killers.

**Figure:**
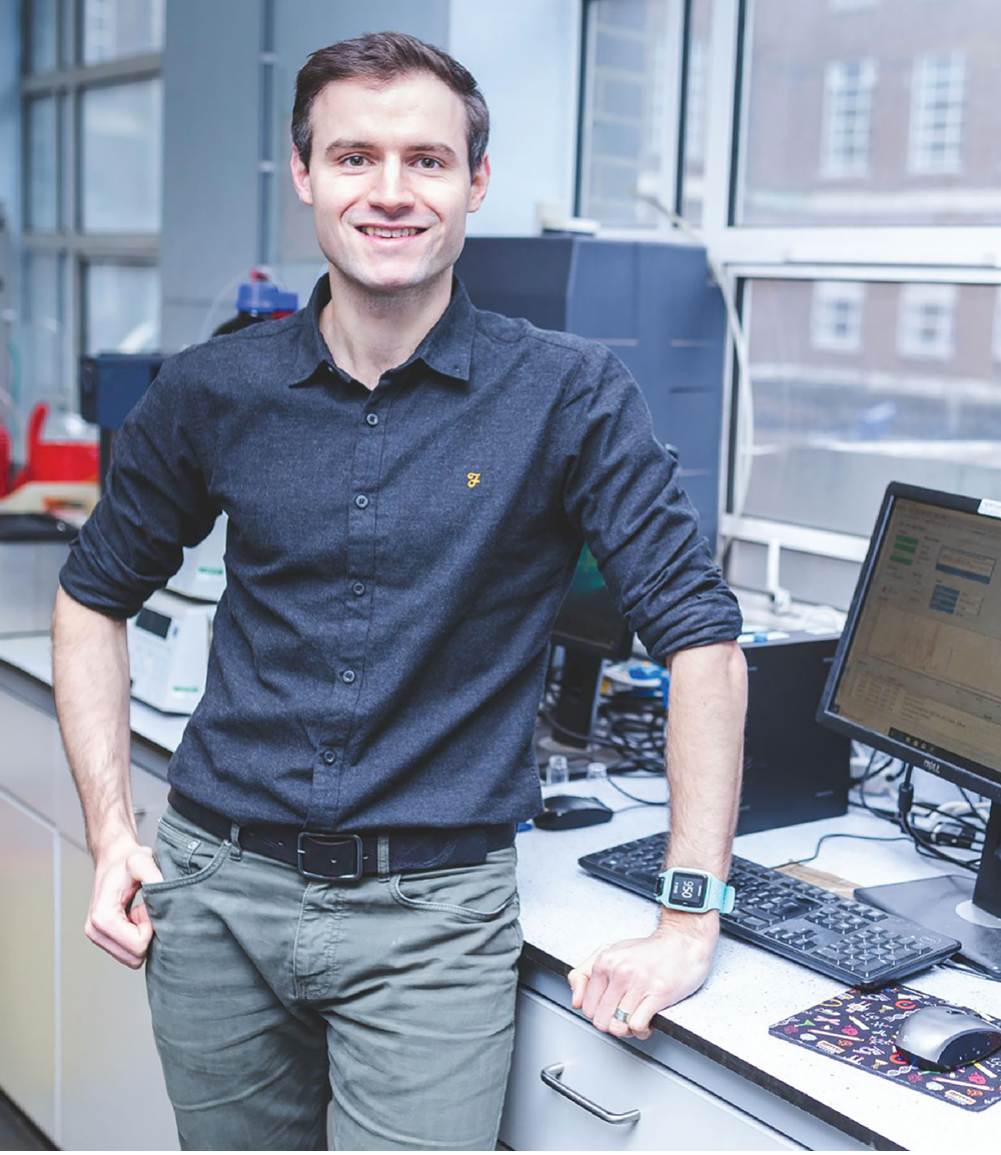
Credit: Daniel Corbett

Stephen Cochrane of Queen’s University Belfast is thinking outside the box. Instead of sending molecules into bacteria, Cochrane designs and builds compounds, like certain peptides, that kill bacteria by binding to the lipids that form their outer surfaces. He says this approach can uncover drug candidates that bacteria have a tougher time developing resistance to because membrane lipids tend to evolve more slowly than internal protein defenses.

With a €1.5 million ($1.6 million) grant from UK Research and Innovation, he plans to lead a research effort dubbed New HOPE (New Approaches to Overcome the Problem of Antimicrobial Resistance) to find membrane-targeted antibiotics. His team of five early-career researchers aims to create tools that will enable chemists to discover and develop membrane-targeted drugs.

Geoffrey Kamadi spoke with Cochrane about why we need a new approach to antibiotics, what sorts of drugs could be out there, and his plan for the 5 years of his project.

## Given the number of different antibiotics sold today, why try to make new ones?

Since the golden age of antibiotics [roughly 1950–1960], we’ve found that resistance has been developing at a faster and faster rate. The UK government did a report in 2014 which predicted that, if resistance continues at the current rate, by 2050 it will cause 300 million premature deaths and cost the global economy over $100 trillion.

We cannot just make antibiotics and then sit back and say, “OK, these will do the job.” We need to be coming up with antibiotics that act via new mechanisms. If you make an antibiotic that is structurally like anything that exists—say, like a β-lactam—it is acting on the same targets. So resistance is going to happen more quickly with those types of compounds. You want something that is new, that cells have not seen before.

## How is your lab’s approach different from previous antibiotics research efforts?

For decades, extensive efforts have been made to develop antibiotics that target proteins inside cells because the types of technologies needed to do a variety of fluorescence-based protein assays are well established. But it is different for cell membrane targets.

A membrane target is not a protein; it is a lipid embedded in a cell membrane. It has a polar headgroup where the antibiotic can latch on. The problem is that there has not been a concerted effort to discover new compounds that target these membrane targets because the tools needed to do that have not been accessible.

And so what my research aims to do is develop new tools, almost like a sieve, to be able to filter through compound libraries and find compounds that bind to these targets. What we are doing is taking a particular lipid target in the cell membrane and using organic chemistry to add a fluorescent reporter to the polyprenyl tail that anchors these targets to the cell membrane. Whenever we identify an antibiotic that binds to the head of a target, the lipid emits a fluorescent signal that we can detect.

These targets are being screened mostly in multiwell plates, but we hope to also develop chemical probes to study processes in cells.

## What kind of antibiotic candidates has your membrane-targeted approach opened the door for?

In general, larger molecules—things typically above 500 Da—cannot pass through cell membranes passively. So if the target is inside the cell, it limits a lot of the different types of structures that we can use as drugs.

Because we do not need to get these compounds through the cell membrane, we do not have to worry about size. We can get much bigger, complex scaffolds that are going to have more parts binding to the target.

When I teach this concept to students, I use the analogy of a giant squid attacking a boat versus a shark attacking a boat. When a shark attacks, there’s only one attachment point, and any mutation or modification of the target blocks binding. In contrast, when a giant squid gets all its arms around a boat, it does not matter if one or two arms are blocked. It is still bound to the boat. Nonribosomal antimicrobial peptides are a major family of compounds that operate like this.

## What are your first steps as you plan to use the new grant?

In the first 1 or 2 years, we are going to develop the chemistry needed to make tools for discovery. We are then going to use the tool kit to do two kinds of broad things.

In collaboration with Foundation Medina in Spain, we will use the tools to screen their natural product libraries. They are world leaders in natural product discovery. Also, our team will be looking at developing a new and much more efficient way to chemically synthesize these membrane targets. Because for us to develop tools, we need to be able to synthesize the targets we are interested in.

In another branch of the research, we are going to look at different enzymes that process or make some of these targets and see if we can use our tools to develop new assays to find inhibitors for those enzymes. So, 2 to 3 years will be tool development, and then years 4 and 5 will be using the tools to find new antibiotics.

## What do you hope to achieve by the end of the 5-year project?

I hope to have a number of new antibiotic candidates that operate by new mechanisms. And by the end of this project, we will develop chemical tools that can be used by researchers around the world to screen for new compounds with membrane targets.

I am very optimistic we will find something new. I cannot say for certain whether that then will become a new antibiotic because of course that is a much different challenge.

But I do not think it is intimidating at all. I really enjoy the work I do. I mean, I am a scientist primarily because I am curious about different things. It is good that my curiosity is piqued by ways to kill bacteria.

## Geoffrey Kamadi is a freelance contributor to

Chemical & Engineering News, *the independent news publication of the American Chemical Society. This interview has been edited for length and clarity.*

